# Diagnostic accuracy of rib fracture detection in forensic post-mortem photon counting CT

**DOI:** 10.1007/s00414-025-03597-w

**Published:** 2025-09-16

**Authors:** Paolo Lombardo, Conny Hartmann, Chantal Fridle, Hendrik von Tengg-Kobligk, Thomas D. Ruder, Wolf-Dieter Zech

**Affiliations:** 1 Institute of Forensic Medicine, University of Bern, Bern, Switzerland; 2 Department of Diagnostic, Interventional and Pediatric Radiology, Inselspital, Bern University Hospital, University of Bern, Bern, Switzerland

**Keywords:** Post-mortem computed tomography, Photon-counting CT, Diagnostic accuracy, Rib fractures

## Abstract

**Background:**

Recently, post-mortem photon-counting CT (PMPCCT) has been introduced to forensic imaging. Compared to conventional energy integrating post-mortem CT (PMCT), PMPCCT enables ultra-high resolution (UHR) imaging, which is particularly suitable for visualizing fine fractures and fissures. These are often found in rib trauma, which is usually relevant in forensic medicine. The aim of this study was to evaluate the diagnostic accuracy of rib fracture detection using PMPCCT compared to PMCT and forensic autopsy.

**Methods:**

A total of *n* = 20 bodies that had suffered blunt chest trauma before death and *n* = 5 non-trauma control cases were investigated. PMCT and PMPCCT were conducted prior to forensic autopsy. Two observers (radiologist and forensic pathologist) analyzed PMCT images (reconstructed with a slice thickness of 0.5 mm in a 512 matrix) and PMPCCT images (reconstructed with slice thicknesses of 0.5 mm and 0.2 mm (UHR) in a 1024 matrix). Imaging diagnosis of rib fractures was compared to autopsy diagnosis as gold standard. The diagnosis of different types of rib fractures assessed at imaging was compared between PMCT and PMPCCT.

**Results:**

Inter-rater agreement between the two observers was good (κ = 0.79). Both PMCT and PMPCCT diagnosed less rib fractures than the autopsy (*n* = 356 fractures). Compared to PMCT, UHR-PMPCCT demonstrated slightly higher sensitivity (e.g., 92% vs. 82% in observer 1), slightly higher specificity (e.g., 97% vs. 95%), and lower positive predictive value (e.g., 75% vs. 82%) for the diagnosis of all rib fractures. Chi-squared tests showed significant differences (p-value < 0.05 in both observers) between UHR-PMPCCT and PMCT for the diagnosis of incomplete rib fractures with visible dehiscence of one cortical line.

**Conclusion:**

Based on the results, it is questionable whether PMPCCT offers additional forensic value over PMCT for the specific forensic issue of diagnosing rib fractures.

## Introduction

In recent decades, energy-integrating post-mortem computed tomography (PMCT) has established itself as a useful adjunct to autopsy in forensic medicine [1–4]. In recent years, the new technique of photon counting detector CT (PCCT) has been increasingly used in certain areas of clinical radiology [5–8]. PCCT uses detectors capable of detecting individual incident photons and their energy levels. The improved detector design leads to a reduction in electronic circuit noise, beam hardening, and metal artifacts. Furthermore, an increase in the contrast-to-noise ratio and spatial resolution can be achieved. Conventional CT can currently achieve slice thicknesses of up to 0.5 mm with a 512 matrix. PCCT enables ultra-high resolution (UHR) of up to 0.2 mm with a 1024 matrix [9–11]. Due to its properties, PCCT is particularly well suited for bone imaging and the detection of fine fractures and fissures [7, 12]. Since fracture detection is usually relevant in forensic medicine, post-mortem PCCT (PMPCCT) appears to offer potential added value in the field of postmortem imaging. Although several PMPCCT studies have been conducted on whole cadavers or single cadaver parts, these are almost exclusively non-forensic studies with a clinical background and therefore protocols optimized for living patients with the lowest possible radiation dosage were used [13–20]. Forensic post-mortem CT, however, typically uses protocols optimized for deceased individuals with much higher radiation doses [21, 22]. Accordingly, optimized studies are needed for forensic PMPCCT [23, 24].

In forensic medicine, rib fractures are frequently encountered as a result of chest trauma or cardiopulmonary resuscitation [25–28]. The detection of rib fractures in forensic PMCT can be of great relevance for reconstructing the nature of the underlying trauma. Furthermore, rib fractures can be associated with findings relevant to the cause of death, such as hemo-pneumothorax [29–33]. However, the diagnosis of rib fractures can be challenging both in autopsy and PMCT [28, 31]. Non-displaced fractures, in particular, can be difficult to detect on PMCT if no fracture line is visible or if no kinking or bending of the rib cortical line is evident. Previous PMCT studies evaluated the diagnostic accuracy of rib fractures compared to autopsy in adults. These studies showed moderate sensitivities and high specificities for diagnosing rib fractures [31, 33]. Given the theoretically improved capabilities of PCCT regarding bone imaging, the question arises as to whether PMPCCT is superior to PMCT for the diagnosis of rib fractures. Therefore, the aim of this study was to evaluate the diagnostic accuracy of rib fracture detection using PMPCCT compared to PMCT and forensic autopsy.

## Methods and materials

### Study design and study subjects

This prospective study was conducted in accordance with the ethical standards of the Declaration of Helsinki and approved by the Cantonal Ethics Committee of Bern (reference number KEK 2023 − 00460). *N* = 25 cadavers (13 males and 12 females, mean age 54 years, mean height 170 cm, mean weight 77 kg) from the Institute of Forensic Medicine Bern were examined. *N* = 20 study subjects suffered blunt chest trauma either because of accidental or suicidal trauma and/or agonal mechanical cardiopulmonary resuscitation (*n* = 6 falls from height (2 of those with cardiopulmonary resuscitation); *n* = 7 traffic accidents (4 of those with cardiopulmonary resuscitation); *n* = 7 cardiopulmonary resuscitations due to intoxications or acute cardiac arrest without prior trauma). *N* = 5 control cases died of acute cardiac arrest and did not suffer trauma or resuscitation at all. All bodies were autopsied. Prior to autopsy, a conventional PMCT scan was conducted at the Institute of Forensic Medicine Bern. Consequently, a second scan using a PMPCCT was performed at the Department of Diagnostic, Interventional and Pediatric Radiology at the University Hospital Bern. Inclusion criteria were age over 18 years and no relevant putrefaction. The post-mortem interval (time between death and autopsy) ranged between 0.5 and 3 days. Before the CT scans, the corpses were stored in cold chambers at 4 °C.

## PMCT and PMPCCT

All CT scans were conducted with the corpses in the supine position in artefact free body bags. For PMCT (Siemens Somatom^®^ X.cite) whole-body scans, and for PMPCCT (Siemens NAEOTOM^®^ Alpha) head and torso scans were performed.

PMCT acquisition and reconstruction parameters were based on current PMCT protocol standards [21, 22]. Tube current Dose modulated 700 ref. mAs; tube voltage 140 kV; collimation 120 × 0.5; pitch 0.25; rotation time 2000 ms. Field of view was adapted to thorax; matrix 512 × 512, thorax reconstruction filter Br 60; reconstructed slice thickness 0.5 mm, increment 0.25 mm.

PMPCCT acquisition and reconstruction parameters were based on previous PMPCCT publications from Zech et al. and Gascho et al. [23, 24]. Tube current Dose modulated 700 ref. mAs; tube voltage 140 kV; collimation 120 × 0.25; pitch 0.25; rotation time 2000 ms. Field of view was adapted to thorax; matrix 1024 × 1024; thorax reconstruction filter Br 60, reconstructed slice thicknesses 0.2 mm UHR (increment 0.1 mm) and 0.5 mm (increment 0.25 mm).

Table 1 shows the average radiation doses administered as well as the average image series data size and contrast-to-noise ratios (CNR) for all PMCT and PMPCCT examinations and reconstructions. CNR was calculated using the methodology described by Huang et al. [34].

**Table 1:** PMCT and PMPCCT average radiation doses administered as well as the average image series data size and contrast-to-noise ratios (CNR) for all image reconstructions

	**PMCT**	**PMPCCT**	
*DLP (mGycm)*	3850	3680	3680
*Effective mAs*	412	405	405
*Slice thickness (mm)*	0.5	0.5	0.2
*Slice distance (mm)*	0.25	0.25	0.1
*Reconstruction kernel*	Br60	Br60	Br60
*Reconstructed FoV (cm)*	35	35	35
*Image series data size (GB)*	0.64	1.75	4.2
*CNR*	54	65	48

## Radiological image analysis

**Fig. 1:**
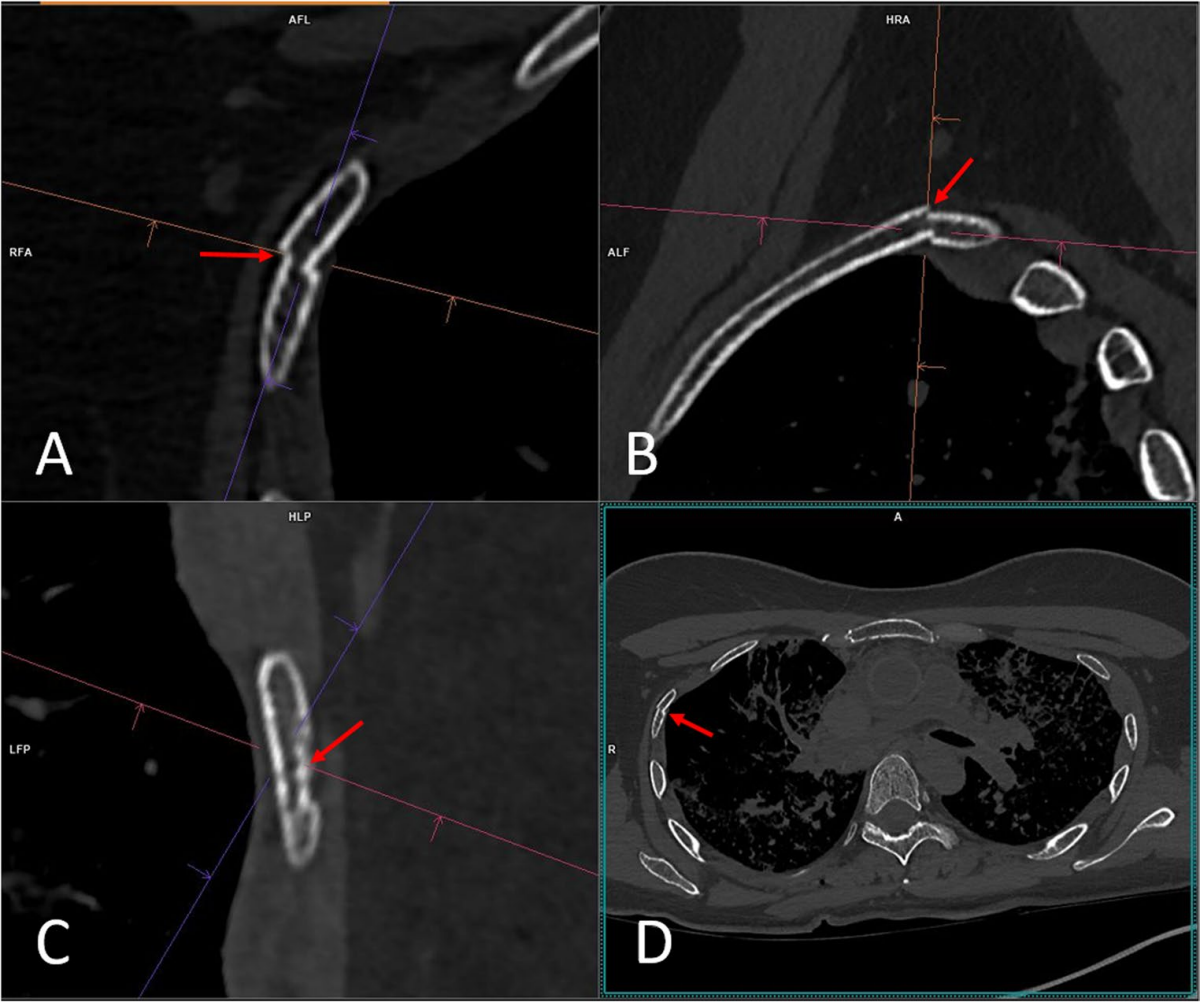
Example of radiological assessment of rib fractures. Assessment was conducted in multiplanar (A-B) and axial (D) reconstruction images by two observers independently side by side and rib by rib in PMCT and PMPCCT. In the case shown, these are PMCT images reconstructed with 0.5 mm slice thickness and a Br60 filter. The images show a fracture (arrows) of the third right rib

PMCT and PMPCCT image analyses were conducted independently by two observers (a board-certified radiologist (observer 1) with 10 years of experience in post-mortem imaging and a board-certified forensic pathologist (observer 2) with 15 years of experience in post-mortem imaging) with a syngo.via^®^ workstation (Siemens). Both observers were blinded to autopsy results. The observers evaluated axial as well as multiplanar MPR reconstruction images of the thorax (Fig. 1). No 3D reconstructions or automated rib fracture software tools were used. The data sets examined included PMCT images reconstructed with a slice thickness of 0.5 mm (512 × 512 matrix, filter Br60), and PMPCCT images reconstructed with a slice thickness of 0.2 mm (UHR) as well as 0.5 mm (Matrix 1024 × 1024,Filter Br60).

Images were evaluated sequentially for the presence and location of bony rib fractures and parasternal cartilage rib fractures rib-by-rib and side-by-side. Sternal fractures were not assesed. The two different CT modalities as well as different slice thicknesses in PMPCCT were viewed with an at least 7-days’ time difference in order to avoid memory bias. If multiple fractures were present in a single rib, these were counted individually. For the bony rib fractures, different fracture types were categorized as follows:

- Complete fractures: Interruption of both cortical lines either displaced (defined as greater than half a rib shaft width) or non-displaced;
- Incomplete fractures: Interruption of only one (either the inner or outer) cortical line with either visible dehiscence (with or without kinking/bending of the unilateral rib cortex) or kinking/bending of one cortical line without visible dehiscence of the cortical line.

## Autopsy

Local authorities ordered forensic autopsies. Board certified forensic pathologists performed the autopsies according to the Recommendation of the Committee of Ministers to Member States of Europe on the harmonization of medico-legal autopsy rules [35] and blinded for PMCT and PMPCCT diagnostic rib fracture results. During the autopsy dissection of the thorax, each intercostal space was completely sliced and each rib was visually inspected, palpated for cortical irregularities and manually tested for rib stability. Visible bone damage and/or instability during manual movement of the rib were considered fractures. The location of each fracture was noted. Single ribs were neither removed nor macerated.

## Statistics

Rib fractures diagnosed at PMCT, PMPCCT and autopsy were compared. Sensitivity, specificity, negative and positive predictive values with 95% confidence intervals (CI) were calculated with the autopsy as gold standard.

Cohen`s kappa values were calculated to determine the inter-observer variability for the post-mortem imaging diagnosis of rib fractures and interpreted as follows: excellent agreement: κ > 0.81; good agreement: κ = 0.61–0.80; moderate agreement: κ = 0.41–0.60; slight agreement: κ = 0.21–0.40; poor agreement: κ = 0–20 [36]. Inter-observer variability was defined as the difference in rib fracture diagnosis made between the two observers. Rib fracture diagnoses in PMCT and PMPCCT were compared using the chi-squared test. P-values < 0.05 were assumed as statistically significant. All calculations were conducted using SPSS (IBM, Version 23.0).

## Results

A total of *n* = 600 ribs from 25 cadavers were examined. Each of the *n* = 20 bodies that suffered blunt chest trauma and/or mechanical cardiopulmonary resuscitation had at least four rib fractures at autopsy. Of the *n* = 5 control cases, no rib fractures were diagnosed at autopsy or imaging.

Contrast-to-noise ratio was highest in the 0.5 mm PMPCCT image series and lowest in the UHR-PMPCCT image series. The data size of the UHR-PMPCCT image series was significantly higher (by a factor of approximately 6.6) than that in the PMCT data series (Table 1).

Table 2 shows the total number of rib fractures diagnosed at autopsy, PMCT and PMPCCT imaging respectively. A total of *n* = 356 rib fractures were diagnosed at autopsy. In PMCT and PMPCCT, both observers diagnosed fewer rib fractures overall than autopsy, with the majority of rib fractures being diagnosed on UHR-PMPCCT. Both observers showed higher false positive rates for rib fractures in the PMPCCT, with the highest rates recorded in the UHR-PMPCCT.

**Table 2:** Numbers of rib fractures diagnosed at autopsy (gold standard), PMCT and PMPCCT.* Numbers of true positive rib fractures, ** Numbers of false positive rib fractures

		***Observer 1***			***Observer 2***		
**Rib No**	**Autopsy**	**PMCT**	**PMPCCT** **0.5 mm / 0.2 mm**		**PMCT**	**PMPCCT** **0.5 mm / 0.2 mm**	
1	12	9	9	10	8	9	9
2	39	35	38	38	34	37	36
3	59	51	53	55	49	52	53
4	55	48	50	51	49	49	50
5	54	44	47	50	45	45	49
6	48	43	42	43	41	40	42
7	37	31	34	35	30	32	35
8	21	17	16	19	16	17	19
9	12	9	10	10	9	11	10
10	9	7	8	9	6	8	8
11	6	3	5	5	2	4	5
12	4	2	2	3	2	2	3
**Total**	**356**	299 (245*, 54**)	314 (251*, 63**)	328 (246*, 82**)	291 (232*, 59**)	306 (242*, 64**)	319 (236*, 83**)

Inter-rater agreement between observer 1 and observer 2 for rib fracture diagnosis was good with a Cohen`s kappa values of κ = 0.79 (95% confidence interval: 0.73–0.84).

Table 3 shows sensitivity, specificity, as well as positive and negative predictive values for PMCT and PMPCCT compared to autopsy for rib fracture diagnosis. UHR- PMPCCT showed higher sensitivity for rib fracture diagnosis than non UHR-PMPCCT (8% higher in observer 1 and 7% in observer 2) and PMCT (10% higher in observer 1 and 9% in observer 2). UHR-PMPCCT showed slightly lower positive predictive values compared to non-UHR PMPCCT (5% less in both observers) and PMCT (7% lower in observer 1 and 6% in observer 2). Specificity and negative predictive values were over 90% in PMCT and PMPCCT for both observers with slightly higher values in PMPCCT.

**Table 3:** Diagnostic performance of PMCT and PMPCCT compared to autopsy as gold standard for rib fracture diagnosis. (*) = 95% confidence intervals

	Sensitivity as % (*)		Positive predictive value as % (*)		Specificity as % (*)		Negative predictive value as % (*)	
	*Observer 1*	*Observer 2*	*Observer 1*	*Observer 2*	*Observer 1*	*Observer 2*	*Observer 1*	*Observer 2*
**PMCT**	82 (78–84)	81 (79–86)	82 (79–85)	80 (78–83)	95 (92–98)	94 (90–97)	94 (88–96)	94 (90–97)
**PMPCCT (0.5 mm)**	84 (82–87)	83 (78–85)	80 (78–83)	79 (76–84)	95 (92–98)	94 (91–96)	94 (91–97)	93 (89–96)
**PMPCCT (0.2 mm)**	92 (88–96)	90 (88–93)	75 (71–78)	74 (70–78)	97 (89–95)	96 (88–94)	97 (92–97)	97 (91–99)

Table 4 shows the number of different types of rib fractures recorded by both observers in PMCT and PMPCCT. The majority of rib fractures observed in imaging were complete fractures. All detected parasternal cartilage rib fractures were complete fractures. In complete displaced rib fracture diagnosis, no differences were found between PMCT and PMPCCT in both observers. For the remaining complete and incomplete rib fracture types, differences in diagnostic numbers were found between UHR-PMPCCT, non-UHR PMPCCT and PMCT. However, the chi-squared tests showed only significant differences between rib fractures with visible dehiscence in UHR-PMPCCT vs. PMCT regarding the different fracture types (Table 5). The remaining fracture types showed no significant differences between PMCT and PMPCCT. However, in these groups, the lowest p-values ​​were found for the UHR-PMPCCT vs. PMCT groups.

**Table 4:** Numbers of different types of rib fractures assessed at PMCT and PMPCCT for both observers

	**Observer 1**			**Observer 2**		
**Rib fractures**	**PMCT**	**PMPCCT (0.5 mm)**	**PMPCCT (0.2 mm)**	**PMCT**	**PMCCT (0.5 mm)**	**PMPCCT (0.2 mm)**
**Complete**						
*non-displaced*	76	79	84	74	76	80
*displaced*	115	115	115	115	115	115
**Total**	**191**	**194**	**199**	**189**	**191**	**195**
**Incomplete**						
dehiscence of cortex	37	47	55	33	44	52
buckle / bending / kinking of cortex	71	73	74	67	71	72
**Total**	**108**	**120**	**129**	**102**	**115**	**124**

**Table 5:** Results for chi-squared tests of rib fracture diagnosis between PMCT and PMPCCT

	**Observer 1**	**Observer 2**
	***p*** **-value **	***p*** **-value**
***Complete rib fractures***		
*non-displaced*		
PMCT vs. PMPCCT (0.5 mm)	0.79	0.86
PMCT vs. PMPCCT (0.2 mm)	0.55	0.66
PMPCCT (0.5 mm) vs. PMPCCT (0.2 mm)	0.74	0.73
*displaced*		
PMCT vs. PMPCCT (0.5 mm)	1	1
PMCT vs. PMPCCT (0.2 mm)	1	1
PMPCCT (0.5 mm) vs. PMPCCT (0.2 mm)	1	1
***Incomplete rib fractures***		
dehiscence of cortex		
PMCT vs. PMPCCT (0.5 mm)	0.25	0.19
PMCT vs. PMPCCT (0.2 mm)	0.04	0.03
PMPCCT (0.5 mm) vs. PMPCCT (0.2 mm)	0.41	0.39
buckle / bending / kinking of cortex		
PMCT vs. PMPCCT (0.5 mm)	0.86	0.72
PMCT vs. PMPCCT (0.2 mm)	0.79	0.65
PMPCCT (0.5 mm) vs. PMPCCT (0.2 mm)	0.93	0.93
***ALL rib fractures***		
PMCT vs. PMPCCT (0.5 mm)	0.39	0.39
PMCT vs. PMPCCT (0.2 mm)	0.09	0.11
PMPCCT (0.5 mm) vs. PMPCCT (0.2 mm)	0.42	0.45

In none of the *n* = 20 cases involving rib fractures would the PMPCCT results have had any forensic consequences, as they did not raise any new forensic questions.

## Discussion

**Fig. 2:**
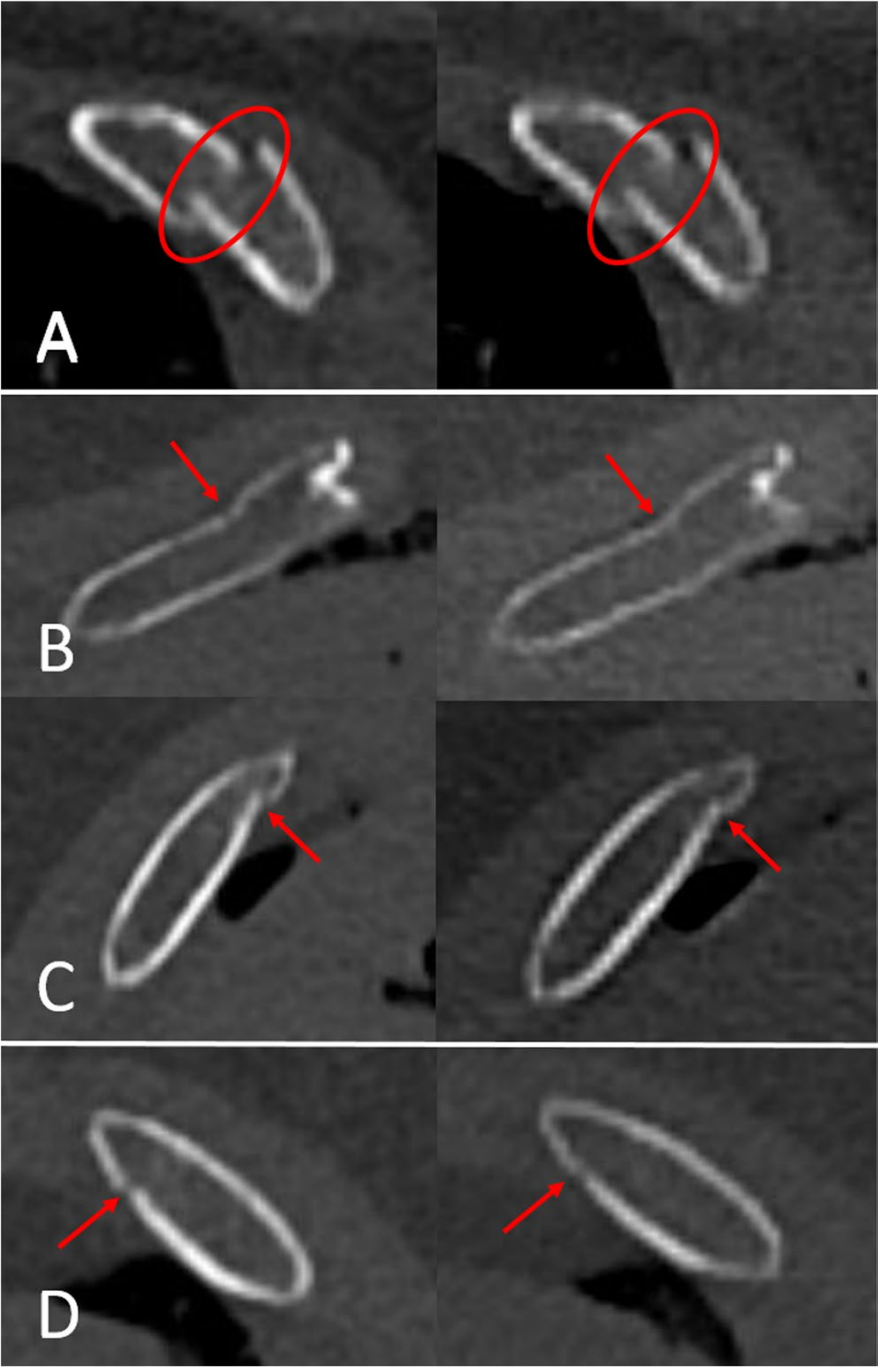
Examples of rib fractures (encirclement in A, arrows in B-D) in UHR-PMPCCT (left side of images) reconstructed at 0.2 mm with a 1024 × 1024 matrix and PMCT (right side of images) reconstructed at 0.5 mm with a 512 × 512 matrix. Notice better visualization of the cortical line structures in UHR-PMPCCT. A: complete fracture. B-D: incomplete fractures with dehiscence of one cortical line

The results of the present study showed that both UHR-PMPCCT and PMCT detected fewer rib fractures than forensic autopsy. However, UHR-PMPCCT provided slightly higher sensitivity than PMCT. Overall, the PMCT results of the study are in line with previous PMCT studies that showed moderate to high sensitivities and overall high specificities for the diagnosis of rib fractures compared to forensic autopsy [31, 33]. In this context it should be noted that conventional energy integrating PMCT, reconstructed with a slice thickness of 0.5 mm and a 512 matrix already produces very high-quality images. Therefore, major diagnostic differences between PMCT and PMPCCT were not expected, especially for displaced rip fractures. However, both radiological observers noted that UHR-PMPCCT provided a better visualization of the rib cortical lines than PMCT (Fig. 2). Rib fractures with slight displacement, bending, or kinking of the cortical lines are relatively easy to detect with conventional PMCT. In contrast, rib fractures in which the fracture ends are non-displaced and the cortical lines exhibit only discrete dehiscence are more difficult or even impossible to diagnose with PMCT. In these cases, PMPCCT partially revealed fracture-related dehiscence that was missed in PMCT. Hence, in the statistical comparison of the different fracture types, PMCT and UHR-PMPCCT only showed significant differences between incomplete rib fractures with dehiscence of the cortical line. These cases may explain the overall higher sensitivity of PMPCCT. However, it was found that even UHR-PMPCCT could not detect all rib fractures identified at forensic autopsy. It can be speculated that these were fractures that may not exhibit displacement of the fracture ends, dehiscence of the cortical lines, or bending or kinking of the cortical lines. A limitation of this study was that autopsy could only determine the presence of fractures but not the exact fracture type as categorized at imaging. The reason for this was that the individual ribs were not removed during the autopsy and therefore could not be macerated. Without maceration, a reliable distinction of the exact rib fracture type cannot be made with autopsy. Thus, no exact fracture types could be compared between autopsy on the one hand and PMCT and PMPCCT on the other. Future studies should be conducted with macerated ribs to accurately compare different fracture types between autopsy, PMCT, and PMPCCT.

In this context, a notable study finding was a slightly lower positive predictive value in the UHR-PMPCCT than in the PMCT. This could be explained, on the one hand, that more false-positive rib fractures were detected in the PMPCCT. On the other hand, it is also conceivable that PMPCCT detected fractures that were missed at autopsy and PMCT. Previous studies discussed that PMCT may detect incomplete fractures that are missed at autopsy [29–33]. The advantage of autopsy over imaging is that the ribs can be moved manually, allowing non-displaced rib fractures to be detected due to instability. However, incomplete rib fractures may not show any instability during movement and thus be missed. In such cases, PMPCCT may be superior to autopsy, but as already mentioned above, this could only be determined with certainty in comparison with macerated ribs.

Previous PMCT studies have shown that software tools for the automatic detection of rib fractures can provide added diagnostic value in post-mortem imaging [30, 33]. Based on the results of the present study, it seems possible that PMPCCT could also benefit from such applications. Future studies should therefore investigate the diagnostic performance of such tools.

In view of the results, the question arises as to whether a potentially minor improvement in the detection of rib fractures in PMPCCT would be relevant at all in forensic practice. In the specific cases of the present study, the results of the PMPCCT did not reveal any new findings of forensic relevance. This is because in all cases there were serial rib fractures and the sequence of events was known with no new findings to be expected. However, in the authors’ view, this does not rule out the possibility that there could be forensic case constellations in which a slightly improved diagnostic accuracy of the PMPCCT could be of relevant significance. This could apply, for example, to cases in which the question arises as to whether chest trauma has occurred at all and no fractures can be identified in the autopsy. If, as discussed above, PMPCCT can indeed represent incomplete rib fractures that are not visible in the autopsy, this could represent a relevant added value. However, as mentioned above, this would require further studies with completely macerated ribs.

Another question that arises based on the results is whether, compared to PMCT, the potentially minor diagnostic improvements of PMPCCT justify the currently still considerably higher costs and significantly higher data volumes. Future studies should therefore investigate the potential added value of PMPCCT for further forensic questions. Comparative studies of special forensic cases in which assessment using PMCT can be particularly difficult, such as decomposed corpses, burned corpses, newborns and infants, strangulation cases, and severely overweight corpses, also appear relevant here.

## Limitations

In addition to the limitations already listed above, further significant limitations of the study must be mentioned:

Overall, only a relatively small number of ribs and rib fractures were examined. Although the sample size was sufficient for statistical analysis, the validity of the study would be increased with a larger sample size.

Both radiological observers had relatively extensive experience, and inter-observer variability was also approximately equal. Previous PMCT studies have shown that the diagnostic accuracy for rib fractures depends significantly on the observers experience [33]. Therefore, different results might be expected for observers with more or less experience. In this context, the results of examinations using automated rib fracture detection software would also be potentially interesting, but these were not carried out in this study.

Because the study design did not allow for blinded analysis of the CT scanner type, both objective and especially subjective measurements may have been subject to observation bias, resulting in an overestimation of PMPCCT and an underestimation of PMCT.

Only one bone reconstruction kernel was used for each CT modality. Previous PMPCCT studies with a clinical background demonstrated, that diagnostic performance may differ between different bone kernels and UHR-PMPCCT reconstructions may not always provide better diagnostic results than non-UHR reconstructions [15, 20]. Therefore, future studies should investigate the diagnostic performance of different reconstruction filters for PMPCCT.

Although the corpses were generally handled with great care, it cannot be completely ruled out that individual rib fractures occurred after the PMCT or PMPCCT due to moving the body from the CT table and transport between the different CTs. Similarly, after imaging and prior to autopsy, the bodies were moved and rotated as part of routine external examination. Here, too, it cannot be ruled out, that isolated rib fractures occurred.

The findings from this study are applicable only to adults, not to children. Pediatric rib fractures are always highly relevant in forensic context. Previous studies have shown that PMCT has added value for the diagnosis of pediatric rib fractures, but assessment is challenging due to the finer and only partially ossified bone structures compared to adults [37, 38]. The results of the present study indicate that UHR-PMPCCT may offer added diagnostic value compared to conventional PMCT, which should be investigated in future studies.

## Conclusion

Compared with autopsy as the gold standard, UHR-PMPCCT showed an overall higher sensitivity and slightly higher specificity for rib fracture diagnosis than conventional PMCT. Based on the results, it is questionable whether PMPCCT offers additional forensic value over PMCT for the specific issue of diagnosing rib fractures.
